# Comparing the Accuracy of Visual and Computerized Onset Detection Methods on Simulated Electromyography Signals with Varying Signal-to-Noise Ratios

**DOI:** 10.3390/jfmk6030070

**Published:** 2021-08-23

**Authors:** Erik Kowalski, Danilo S. Catelli, Mario Lamontagne

**Affiliations:** 1 Human Movement Biomechanics Laboratory, School of Human Kinetics, Faculty of Health Sciences, University of Ottawa, Ottawa, ON K1N 6N5, Canada; ekowa053@uottawa.ca (E.K.); danilo.catelli@uottawa.ca (D.S.C.); 2 Division of Orthopeadic Surgery, The Ottawa Hospital, Ottawa, ON K1H 8L6, Canada

**Keywords:** approximated generalized likelihood ratio, double threshold, visual detection, muscle activity, surface EMG onset

## Abstract

Electromyography (EMG) onsets determined by computerized detection methods have been compared against the onsets selected by experts through visual inspection. However, with this type of approach, the true onset remains unknown, making it impossible to determine if computerized detection methods are better than visual detection (VD) as they can only be as good as what the experts select. The use of simulated signals allows for all aspects of the signal to be precisely controlled, including the onset and the signal-to-noise ratio (SNR). This study compared three onset detection methods: approximated generalized likelihood ratio, double threshold (DT), and VD determined by eight trained individuals. The selected onset was compared against the true onset in simulated signals which varied in the SNR from 5 to 40 dB. For signals with 5 dB SNR, the VD method was significantly better, but for SNRs of 20 dB or greater, no differences existed between the VD and DT methods. The DT method is recommended as it can improve objectivity and reduce time of analysis when determining EMG onsets. Even for the best-quality signals (SNR of 40 dB), all the detection methods were off by 15–30 ms from the true onset and became progressively more inaccurate as the SNR decreased. Therefore, although all the detection methods provided similar results, they can be off by 50–80 ms from the true onset as the SNR decreases to 10 dB. Caution must be used when interpreting EMG onsets, especially on signals where the SNR is low or not reported at all.

## 1. Introduction

Electromyography (EMG) can be a valuable tool in measuring skeletal muscle electrical output during physical activities and provides easy access to the physiological processes that cause muscles to generate force in order to produce movement [1]. EMG is evaluated clinically and in research settings to diagnose neurological and neuromuscular problems. It is a valuable tool involved in the fields of biomechanics, motor control, neuromuscular physiology, postural control, and physical therapy [2]. Often, the EMG signal is evaluated in the time domain and in the spectral domain. In the spectral domain, mean and median frequencies are evaluated to provide insight into the muscle biochemistry, contraction characteristics [3,4], and muscle fatigue [5]. Common parameters used in the time domain include the root mean square, the average rectified value, and the peak linear envelope [1]. However, it is often necessary to understand when the muscle is activated (on/off), which requires the detection of the muscle onsets and offsets.

The onset of muscle activity is required in many research and medical fields. Various medical fields, such as orthopedics, physiatry, and neurology use EMG in their clinical evaluations [6,7,8,9]. For example, it was identified that patients after a total knee replacement surgery had prolonged activation of their rectus femoris and hamstrings muscles, caused by an earlier onset and later offset of these muscles. This muscle activation pattern after a total knee replacement is associated with a stiff knee pattern, which is a compensatory strategy patients adapt to provide better control of the knee joint while walking [6].

Onset of muscle activity and its detection are accomplished by a variety of different methods including computerized methods [10,11,12] or through visual detection (VD) [13,14,15]. Accurately detecting the onset can be difficult as the EMG signal is sensitive to different types of noise [16]. When the EMG signal has a low signal-to-noise ratio (SNR), the ability to accurately detect the onset becomes compromised [11]. 

Computerized detection methods were created to produce a reliable way to determine muscle onsets. Some commonly used methods include the approximated generalized likelihood ratio (AGLR) [17] and the double threshold (DT) detector [18]. Despite having been created decades ago, these methods are still routinely used [19,20]. Both methods have been shown to perform well at detecting muscle onsets [21], and their performance has only improved with the addition of various modifications, such as conditioning the signal with a Teager–Kaiser energy operator [14]. Although computerized detection methods perform well with good-quality, high-SNR EMG signals [21], researchers need to visually verify these onsets, especially when the SNR is low.

In general, VD is considered accurate [22], but in addition to being time-consuming, its reliability has been questioned due to its poor reproducibility due to both human error and variability between researchers [12]. To show reliability in their onset measurement, researchers can repeat the onset measurements after a set period (e.g., several days or week later) or have multiple individuals take these measurements and report intra- and interrater reliability, respectively. This highlights measurement reliability; however, there is no indication if these measurements are valid or improve with experience.

Trying to simulate a signal is not as simple as generating a batch of noise followed by a region of higher amplitude since it needs to adhere to the same convolutions as a real signal with similar frequency spectra. EMG signals are complex waveforms which vary in frequency, amplitude, and phase angle due to many intrinsic factors [2]. A method of simulated EMG (_SIM_EMG) was described which created signals that were not only visually similar, but also had comparable distributions in frequency domains [23]. Unlike the simple method which either varied the variance level in a white noise sequence [17] or modulated a Gaussian sequence with a truncated Gaussian wave [18], this method includes physiological aspects such as the firing rate of motor units (MUs) and their impulse responses. This method accounts for many aspects of the signal to be precisely controlled, including relaxation and contraction levels, initial relaxation period, transient relaxation–contraction period, contraction period, transient contraction–relaxation period, return to the relaxation condition period, and the SNR, by adding a white noise to the signal [23]. Controlling all these factors would allow for a reliable training tool which may improve VD of real EMG signals.

While computerized detection methods will remain the primary onset detection methods used by researchers due to their consistency and reliability, their performance drops when signal quality is not ideal [21]. VD of EMG signals will always remain there, whether to visually inspect any automated onset detections or detect onsets of low SNR signals. The purpose of this study was to compare computerized and visual detection in determining the onset of _SIM_EMG signals. Based on the previous research [21], we hypothesized that on signals with low SNR (10 dB), visual detection is superior, but at higher SNR, computerized detection methods are more accurate.

## 2. Materials and Methods

### 2.1. Signal Simulation

EMG signals were simulated using previously reported methods [23]. A raw (without noise) signal is created by the convolution of two features: the random process $p\left(t\right)$ that promotes the temporal profile of the MU action potential trains (MUAPTs) and $W_{\lambda },n\left(t\right),$ the impulse of the MU. The raw _SIM_EMG $y_{1}\left(t\right)$ can be written as follows:

(1) $$y_{1}\left(t\right)=p\left(t\right)\times W_{\lambda },n\left(t\right)\;$$

The impulse $W_{\lambda },n\left(t\right)$ was modeled using the first-order Hermite–Rodrigues function [24]:

(2) $$W_{\lambda },n\left(t\right)=\frac{1}{\sqrt{2^{n}n!}}H_{n}\left(\frac{t}{n}\right)\frac{1}{\sqrt{\pi }\lambda }e^{-t^{2}/\lambda }$$

where *λ* is the constant scale factor, *t* is time, and *H_n_* is the first-order (*n* = 1) Hermite function:

(3) $$H_{n}\left(\frac{t}{n}\right)=\frac{2t}{n}$$

The temporal profile of the MUAPTs was based off both the firing rates and the number of recruited motor units modeled using the following piece-wise function:

(4) $$f\left(t\right)=\left\{\begin{array}{c} RL,\\ CL+\left(RL-CL\right)e^{-6\left(t-t_{r}\right)/t_{s}},\\ CL,\\ RL+\left(CL-RL\right)e^{-6\left(t-t_{r}-t_{s}-t_{c}\right)/t_{r}},\\ RL, \end{array}\right.\;\begin{array}{c} 0\leq t<t_{r}\\ t_{r}\leq t<t_{r}+t_{s}\\ t_{r}+t_{s}\leq t<t_{r}+t_{s}+t_{c}\\ t_{r}+t_{s}+t_{c}\leq t<t_{r}+t_{s}+t_{c}+t_{d}\\ t_{r}+t_{s}+t_{c}+t_{d}\leq t<T \end{array}$$

*RL* and *CL* are associated with the relaxed and contracted levels of the muscle, respectively. Constants *t_r_*, *t_s_*, *t_c_*, and *t_d_* were used to delimit the instants in time correlated to the muscle activities representing the initial relaxation period, transient relaxation–contraction, contraction period, transient contraction–relaxation, and return to the relaxation condition [23].

A random variable α was presented from a uniform distribution and was compared to each of the discrete timepoints over the temporal profile to define a random process denoted by $r\left(t\right):$

(5) $$r\left(t\right)=\left\{\begin{array}{c} 0\\ N\left(0,1\right) \end{array}\;\begin{array}{c} \alpha \;\left(t\right)<f\left(t\right)\\ \alpha \;\left(t\right)>f\left(t\right) \end{array}\right.$$

where $N\left(0,1\right)$ is drawn from a Gaussian distribution of mean zero and variance one and $f\left(t\right)$ is the value of the temporal profile which ranged from zero to one. The temporal profile of the MUAPTs themselves, $p\left(t\right)$, was created by summation of these random processes $r\left(t\right)$ for all the firing MUs denoted by integer *γ*:

(6) $$p\left(t\right)=\overset{\gamma }{\underset{i=1}{\sum }}r_{i}\left(t\right)$$

Once $W_{\lambda ,n}\left(t\right)$ and $p\left(t\right)$ were convolved to achieve the raw _SIM_EMG $y_{1}\left(t\right)$, a white noise process $n\left(t\right)$ was added to generate the final _SIM_EMG signal $y\left(t\right)$, where $\sigma_{s}^{2}$ was the variance of the raw _SIM_EMG signal.

(7) $$n\left(t\right)=N\left(0,\;\frac{\sigma_{s}^{2}}{10^{SNR/10}}\right)$$

This noise represented the intrinsic noise associated with acquisition and allowed for the configuration of a desired SNR. The final (with noise) _SIM_EMG signal $y\left(t\right)$ was summarized using this equation:

(8) $$y\left(t\right)=\;\left(W_{\lambda ,n}\left(t\right)\;*\;p\left(t\right)\right)+n\left(t\right)$$

### 2.2. Comparison of Onset Detection Methods

**Figure 1:**
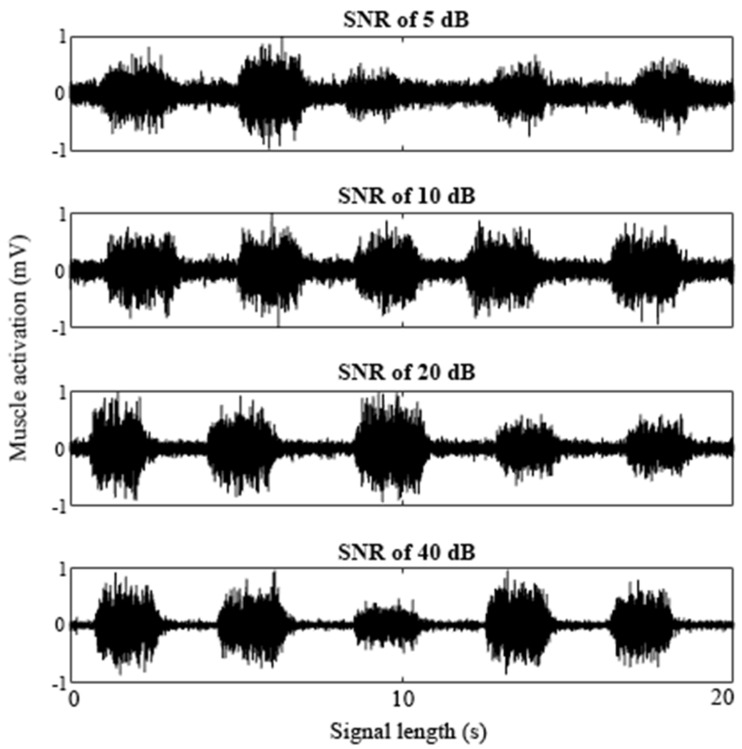
A sample of _SIM_EMG signals with the SNR of 5, 10, 20, and 40 dB. Each _SIM_EMG signal contained five bursts of muscle activity.

Using the methods described above, eight signals, each with five bursts of muscle activity, were simulated to create signals with SNRs of 5, 10, 20 and 40 dB (Figure 1). Two signals of each SNR condition were created to have a total of 10 bursts of activity at each SNR. For each burst, the lengths in time correlated to the muscle activities, RL, and CL were all controlled and varied for each burst. The onset was defined by the initial relaxation period and signaled the beginning of the transient relaxation–contraction period. This value was programmed into the signal and was therefore known, so the true onset was known for each burst of _SIM_EMG.

Two automated detection methods were used, the AGLR [17] and the DT [18]. Custom-made Matlab (MathWorks, Natick, MA, USA) graphical user interfaces (GUIs) were created to implement both detection methods against the 10 _SIM_EMG signals. For the AGLR method, a sliding window (*L*) of 1000 points and the decision threshold (*h*) of 125 were used. For the DT method, the number of samples observed (*m*) was set to 5, the second threshold (*r_o_*) was set to 1, and the probability of false alarm (*P_fa_*) was set to 0.05. Both computerized detection methods were performed on the 10 _SIM_EMG signals three times for a total of 120 onset detections for each method.

Eight individuals with at least 1 year of EMG VD experience (7.1 ± 8.8 years) participated in the study and completed two VD sessions separated by one week. This cohort included seven graduate students and one faculty member. Using the training tool, the participants completed three sets of 10 _SIM_EMG signals at four different SNRs for a total of 120 onset selections. Feedback between their selected onset and the true onset was visually provided at the end of each session.

### 2.3. Graphical User Interface

**Figure 2:**
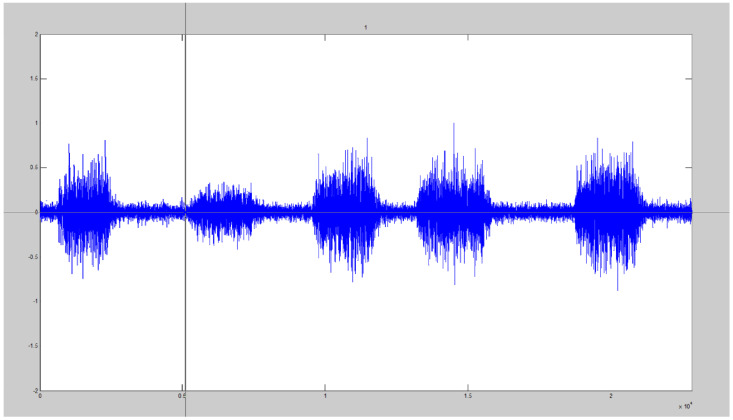
Visual detection GUI with the cursor displayed which allowed participants to select the onset location of five bursts of _SIM_EMG signals.

A custom-made Matlab graphical user interface (GUI) (2017b, Natick, MA, USA) was created which presented eight _SIM_EMG signals in a random order and required the participants to self-select the onset location of five bursts for each signal (Figure 2). The toolbox allowed the participants to make their selection within 1 ms of accuracy.

### 2.4. Statistical Design

The absolute difference between the true onset and the selected onset was determined for each detection method to consider when the onset was either over- or under-estimated. Statistical comparisons were tested with the statistical package SPSS (v22, IBM Corp, Armonk, NY, USA). A Wilcoxon signed-rank test was conducted to determine the effect of the visual detection training tool between the two sessions on the absolute difference between the true onset and the selected onset at various SNRs. A Mann–Whitney *U* test was run to determine if there were differences between the onset detection methods regarding the absolute difference between the true onset and the selected onset at various SNRs. Statistical significance was achieved with *p* ≤ 0.05.

## 3. Results

**Figure 3:**
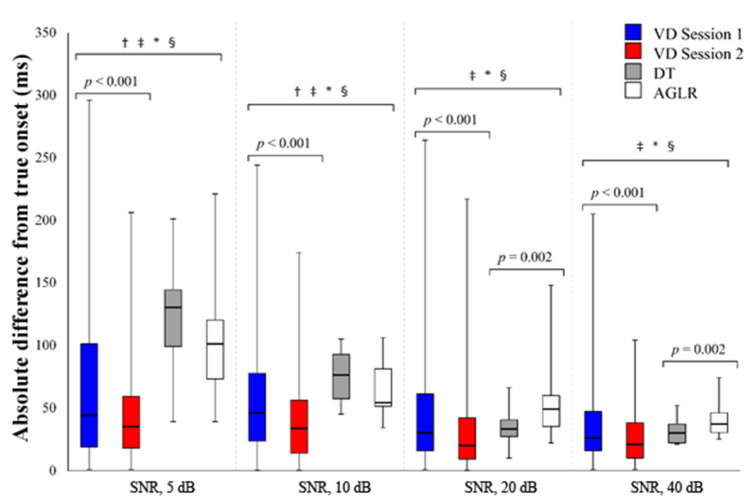
Absolute difference from the true onset (ms) for the different onset detection methods with signal-to-noise ratios of 5, 10, 20, and 40 dB. VD = visual detection; DT = double threshold; AGLR = approximated general likelihood ratio; † represents a significant (*p* < 0.05) difference between VD session 1 and the DT; ‡ represents a significant (*p* < 0.05) difference between VD session 1 and the AGLR; * represents a significant (*p* < 0.05) difference between VD session 2 and the DT; § represents a significant (*p* < 0.05) difference between VD session 2 and the AGLR.

Absolute differences from the true onset (ms) for the different onset detection methods (VD sessions 1 and 2, as well as the DT and the AGLR) with four SNRs are displayed in Figure 3.

### 3.1. Differences between the Two VD Sessions

The second VD session elicited a significant median decrease in the absolute difference of the selected onset from the true onset compared to the first VD session at all SNRs. At the SNR of 5 dB, VD session 2 (35 ms) had a significant decrease compared to VD session 1 (44 ms), z = −5.073, *p* < 0.001. At the SNR of 10 dB, VD session 2 (33.5 ms) had a significant decrease compared to VD session 1 (46 ms), z = −5.209, *p* < 0.001. At the SNR of 20 dB, VD session 2 (20 ms) had a significant decrease compared to VD session 1 (30 ms), z = −5.715, *p* < 0.001. At the SNR of 40 dB, VD session 2 (21 ms) had a significant decrease compared to VD session 1 (26 ms), z = −5.133, *p* < 0.001.

### 3.2. Differences between All the Onset Detection Methods

During the 5 dB SNR condition, both VD sessions had a significant median decrease in the absolute difference of the selected onset compared to both automated detection methods. VD session 1 (44 ms) was significantly lower than the DT (130 ms), z = −5.110, *p* < 0.001, and the AGLR (101 ms), z = −3.884, *p* < 0.001. VD session 2 (35 ms) was significantly lower than the DT (130 ms), z = −7.253, *p* < 0.001, and the AGLR (101 ms), z = −6.100, *p* < 0.001.

During the 10 dB SNR condition, both VD sessions had a significant median decrease in the absolute difference of the selected onset compared to both automated detection methods. VD session 1 (46 ms) was significantly lower than the DT (76 ms), z = −3.574, *p* < 0.001, and the AGLR (54 ms), z = −2.341, *p* < 0.001. VD session 2 (33.5 ms) was significantly lower than the DT (76 ms), z = −5.686, *p* < 0.001, and the AGLR (54 ms), z = −4.536, *p* < 0.001.

During the 20 dB SNR condition, VD session 1 (30 ms) was significantly lower than the AGLR method (49 ms), z = −2.826, *p* = 0.005. VD session 2 (20 ms) was significantly lower than the DT (33 ms), z = −2.635, *p* = 0.008, and the AGLR (49 ms), z = −4.838, *p* < 0.001. The DT (33 ms) was significantly lower than the AGLR method (49 ms), z = −3.058, *p* = 0.002.

During the 40 dB SNR condition, VD session 1 (26 ms) was significantly lower than the AGLR method (37 ms), z = −2.750, *p* = 0.006. VD session 2 (21 ms) was significantly lower than the DT (30 ms), z = −2.616, *p* = 0.009, and the AGLR (37 ms), z = −4.624, *p* < 0.001. The DT (30 ms) was significantly lower than the AGLR method (47 ms), z = −3.108, *p* = 0.002.

## 4. Discussion

The aim of this study was to compare the various detection methods in determining the onsets of _SIM_EMG signals. Our hypothesis was partially correct as the VD methods were significantly more accurate during the 5 and 10 dB SNR conditions. However, at higher SNRs, only after the second session the participants had a lower median absolute difference from the true onset compared to both the DT and AGLR methods (Figure 3).

Noise in EMG signals comes from a variety of sources, including inherent noise generated from electronic equipment, movement artifacts, electromagnetic noise, crosstalk from neighboring muscles, electrocardiographic artifacts, internal noise, or inherent instability of the EMG signal itself [25]. However, EMG signals are often processed and filtered prior to analysis to help remove much of this noise. 

When the SNR is higher, the signal and the baseline noise are more discernible. Regardless of whether VD or automated methods are used, detection accuracy is improved (Figure 3). Of course, signals with higher SNR are ideal, but this is not always feasible. In individuals with Duchenne muscular dystrophy, EMG SNR was around 2 dB [26], which can severely impact onset detection and negatively impact the design of EMG-based control interfaces for assistive technologies.

Muscle onsets and offsets are often used in conjunction with biomechanical variables to provide information on the physiological processes that cause muscles to generate force in order to produce movement [1]. However, an electromechanical delay of 30 and 100 ms exists between the onset of electrical activity and measurable tension [27]. It is believed that the time required to stretch the series elastic component represents the major portion of the measured delay, which is why force development can occur more rapidly in eccentric muscle activities [27]. All the detection methods were within this 30 and 100 ms window of electromechanical delay when SNRs were ≥10 dB, which further improved to under 50 ms when SNRs improved to ≥20 dB.

Caution is needed when interpreting the VD results. Although the VD methods were significantly lower, the interquartile range of the VD sessions was larger compared to both the DT and AGLR for all SNRs ≥ 10 dB. Computerized detection methods have the advantage of increased objectivity of analysis, reduced time requirements, and fewer skill requirements for researchers [13]. VD adds a subjective element when selecting the onsets, and the large range of the data for all SNRs is evidence of this. However, further training on simulated signals may further improve a researcher’s selections at the various SNRs.

There are some limitations to this study that should be addressed. First, the sample size of our study was only nine participants. Although there was a general improvement between the two sessions separated by one week, further research should include more participants and a greater number of sessions to determine how many sessions would be necessary to achieve a ceiling effect. The second limitation of this study was that feedback was only provided at the end of the session and not after each onset selection. Having immediate feedback may be beneficial for improving learning outcomes, so future improvements to the GUI could include this feature. Lastly, it was mentioned earlier that signal conditioning strategies such as the Teager–Kaiser energy operator can improve onset detection. This was not integrated into the GUI for testing either the VD or computerized methods. Different togglable features, such as signal condition strategies, could be included in the GUI to educate users on various methods they could use to assist in selecting the onsets. By including various strategies, users could select their preferred method and incorporate it into the research on real EMG signals.

## 5. Conclusions

In conclusion, this study was successful in showing that the concept of an EMG onset training tool can improve a researcher’s VD of signal onset. VD and DT methods provided similar onsets (within 5 ms) of each other for all signals with the SNR of 20 dB or greater. To improve objectivity and reduce time of analysis, the DT detection method is recommended for signals with the SNR of 20 dB or greater. Caution must be taken when examining onsets of EMG signals when the SNR is low (≤10 dB) or when not reported at all, as the indicated onset may be 50 ms or more away from the true onset, depending on the detection method used. The VD methods were more accurate at low SNRs; however, the use of computerized detection methods is still recommended when signal quality is better due to their lower variability and quicker implication.

## Availability and Requirements

**Project name:** EMG onset training using simulated signals

**Project link:** https://github.com/ekowa053/EMG-Onset-Training-Using-Simulated-Signals

**Programming language**: Matlab

**License:** Apache License, Version 2.0

## References

[1] De Luca C.J. (1997). The Use of Surface Electromyography in Biomechanics. J. Appl. Biomech..

[2] Raez M.B.I., Hussain M.S., Mohd-Yasin F. (2006). Techniques of EMG signal analysis: Detection, processing, classification and applications. Biol. Proced. Online.

[3] Beck T.W., Housh T.J., Johnson G.O., Cramer J.T., Weir J.P., Coburn J.W., Malek M.H. (2007). Does the frequency content of the surface mechanomyographic signal reflect motor unit firing rates? A brief review. J. Electromyogr. Kinesiol..

[4] Catelli D.S., Kuriki H.U., Polito L.F., Azevedo F.M., Negrão-Filho R.F., Alves N. (2011). Patellofemoral pain syndrome: Electromyography in a frequency domain analysis. J. Phys. Conf. Ser..

[5] Cifrek M., Medved V., Tonković S., Ostojić S. (2009). Surface EMG based muscle fatigue evaluation in biomechanics. Clin. Biomech..

[6] Benedetti M.G., Catani F., Bilotta T.W., Marcacci M., Mariani E., Giannini S. (2003). Muscle activation pattern and gait biomechanics after total knee replacement. Clin. Biomech..

[7] D’Apuzzo F., Minervini G., Grassia V., Rotolo R.P., Perillo L., Nucci L. (2021). Mandibular Coronoid Process Hypertrophy: Diagnosis and 20-Year Follow-Up with CBCT, MRI and EMG Evaluations. Appl. Sci..

[8] Fang C., He B., Wang Y., Cao J., Gao S. (2020). EMG-Centered Multisensory Based Technologies for Pattern Recognition in Rehabilitation: State of the Art and Challenges. Biosensors.

[9] Nazmi N., Rahman M.A.A., Yamamoto S., Ahmad S.A., Zamzuri H., Mazlan S.A. (2016). A Review of Classification Techniques of EMG Signals during Isotonic and Isometric Contractions. Sensors.

[10] Liu J., Ying D., Rymer W.Z., Zhou P. (2015). Robust Muscle Activity Onset Detection Using an Unsupervised Electromyogram Learning Framework. PLoS ONE.

[11] Merlo A., Farina D., Merletti R. (2003). A fast and reliable technique for muscle activity detection from surface EMG signals. IEEE Trans. Biomed. Eng..

[12] Tenan M.S., Tweedell A.J., Haynes C.A. (2017). Analysis of statistical and standard algorithms for detecting muscle onset with surface electromyography. PLoS ONE.

[13] Hodges P.W., Bui B.H. (1996). A comparison of computer-based methods for the determination of onset of muscle contraction using electromyography. Electromyogr. Mot. Control Electroencephalogr. Clin. Neurophysiol..

[14] Solnik S., Rider P., Steinweg K., DeVita P., Hortobágyi T. (2010). Teager-Kaiser energy operator signal conditioning improves EMG onset detection. Eur. J. Appl. Physiol..

[15] Van Boxtel G.J., Geraats L.H., Van den Berg-Lenssen M.M., Brunia C.H. (1993). Detection of EMG onset in ERP research. Psychophysiology.

[16] Lee A.S., Cholewicki J., Reeves N.P. (2007). The effect of background muscle activity on computerized detection of sEMG onset and offset. J. Biomech..

[17] Staude G., Wolf W. (1999). Objective motor response onset detection in surface myoelectric signals. Med. Eng. Phys..

[18] Bonato P., D’Alessio T., Knaflitz M. (1998). A statistical method for the measurement of muscle activation intervals from surface myoelectric signal during gait. IEEE Trans. Biomed. Eng..

[19] Bar-On L., Kalkman B.M., Cenni F., Schless S.-H., Molenaers G., Maganaris C.N., Bass A., Holmes G., Barton G.J., O’Brien T.D. (2018). The Relationship Between Medial Gastrocnemius Lengthening Properties and Stretch Reflexes in Cerebral Palsy. Front. Pediatr..

[20] Di Nardo F., Strazza A., Mengarelli A., Cardarelli S., Tigrini A., Verdini F., Nascimbeni A., Agostini V., Knaflitz M., Fioretti S. (2019). EMG-Based Characterization of Walking Asymmetry in Children with Mild Hemiplegic Cerebral Palsy. Biosensors.

[21] Staude G., Flachenecker C., Daumer M., Wolf W. (2001). Onset Detection in Surface Electromyographic Signals: A Systematic Comparison of Methods. EURASIP J. Adv. Signal Process..

[22] Walter C.B. (1984). Temporal quantification of electromyography with reference to motor control research. Hum. Mov. Sci..

[23] Rosa I.d.G., Garcia M.A.C., de Souza M.N. (2008). A novel electromyographic signal simulator for muscle contraction studies. Comput. Methods Programs Biomed..

[24] Conte L.R.L., Merletti R., Sandri G.V. (1994). Hermite expansions of compact support waveforms: Applications to myoelectric signals. IEEE Trans. Biomed. Eng..

[25] Chowdhury R., Reaz M., Ali M., Bakar A., Chellappan K., Chang T. (2013). Surface Electromyography Signal Processing and Classification Techniques. Sensors.

[26] Lobo-Prat J., Janssen M.M.H.P., Koopman B.F.J.M., Stienen A.H.A., De Groot I.J.M. (2017). Surface EMG signals in very late-stage of Duchenne muscular dystrophy: A case study. J. NeuroEngineering Rehabil..

[27] Cavanagh P.R., Komi P.V. (1979). Electromechanical delay in human skeletal muscle under concentric and eccentric contractions. Eur. J. Appl. Physiol. Occup. Physiol..

